# Household airborne endotoxin associated with asthma and allergy in elementary school-age children: a case–control study in Kaohsiung, Taiwan

**DOI:** 10.1007/s11356-020-07899-x

**Published:** 2020-03-25

**Authors:** Yu-Chuan Yen, Chun-Yuh Yang, Tsu-Nai Wang, Pei-Chun Yen, Chi-Kung Ho, Kristina D. Mena, Tzu-Chi Lee, Kang-Shin Chen, Yuan-Chung Lin, Pei-Shih Chen

**Affiliations:** 1 Department of Public Health, College of Health Science, Kaohsiung Medical University, Kaohsiung, Taiwan; 2 Epidemiology, Human Genetics, and Environmental Sciences, School of Public Health, University of Texas Health Science Center at Houston, Houston, TX USA; 3 Department of Health Promotion and Health Education, National Taiwan Normal University, Taipei, Taiwan; 4 Institute of Environmental Engineering, College of Engineering, National Sun Yat-Sen University, Kaohsiung, Taiwan; 5 Department of Medical Research, Kaohsiung Medical University Hospital, Kaohsiung, Taiwan; 6 Research Center for Environmental Medicine, Kaohsiung Medical University, Kaohsiung, Taiwan

**Keywords:** Airborne endotoxin, Bacterial bioaerosol, Fungal bioaerosols, Asthma, Allergy, Children

## Abstract

To evaluate the association between the presence of asthma and allergy, and airborne endotoxin in homes of school-age children in Kaohsiung City, Taiwan, with a case–control study design by matching the age and class exposure. Data collection of home visits included an interviewer-administered questionnaire and air sampling of participants’ homes for endotoxin, bacteria, and fungi, as well as temperature and relative humidity measurements. Endotoxin was detected in all air samples with a median value of 0.67 EU m^−3^. In the adjusted logistic regression model, household airborne endotoxin was associated with higher prevalence of asthma and allergy; OR = 4.88 (95% CI 1.16–20.55) for Q3 (between 0.67 and 1.97 EU m ^−3^) vs. Q1 (< 0.31 EU m ^−3^), with statistical significance. Airborne fungi were associated with higher prevalence of asthma and allergy; OR = 4.47 (95% CI 1.13–17.69) for Q3 (between 314 and 699 CFU m ^−3^) vs. Q1 (< 159 CFU m ^−3^) in adjusted logistic regression models. Airborne endotoxin and fungi were significantly associated with children’s asthma and allergy.

**Electronic supplementary material:**

The online version of this article (10.1007/s11356-020-07899-x) contains supplementary material, which is available to authorized users.

## Introduction

Endotoxin is the lipopolysaccharide component of the cell wall of Gram-negative bacteria that triggers the release of many host mediators of the immune response (Rennie et al. 2012). Endotoxin inhalation in adults in laboratory settings has been shown to induce the hallmarks of asthma: bronchoconstriction, airway inflammation, and bronchial hyperresponsiveness (Kline et al. 1999; Sandström et al. 1992). Previous studies demonstrated that exposure to high levels of endotoxin through inhalation was correlated to some occupational lung diseases among the cotton worker and dairy parlor workers (Ghani et al. 2016; Lai et al. 2014; Nonnenmann et al. 2017; Smit et al. 2008).

In addition to adult exposure in the workplace, non-occupational exposure, especially for schoolchildren, is also of concern to the public, because schoolchildren are more susceptible to respiratory effects than adults. Endotoxin in dust samples was evaluated as an exposure indicator for many epidemiologic studies examining the relationship between endotoxin and schoolchildren’s asthma and allergy, and the results were controversial (Ege et al. 2007; Gehring et al. 2002; Gehring et al. 2008; Leung et al. 2010; Tavernier et al. 2005, 2006). However, dust endotoxin is poorly correlated with airborne endotoxin levels (*r* < 0.34) (Barnig et al. 2013; Mazique et al. 2011; Park et al. 2001; Singh et al. 2011; Sohy et al. 2005). In addition, a stronger association of wheezing with airborne endotoxin was observed than wheezing with dust endotoxin (Horick et al. 2006).

To date, only five studies explored the association between airborne endotoxin exposure and health effects of schoolchildren (Delfino et al. 2015; Hoopmann et al. 2006; Lai et al. 2015; Matsui et al. 2013; Rabinovitch et al. 2005). All of them aimed at asthmatic schoolchildren, and focused on asthma exacerbations, including increase of asthmatic symptoms, decreased lung functions, etc. Therefore, the purpose of this study was to investigate the association between the presence of asthma and allergy, and airborne endotoxin concentration in the homes of school-age children in Kaohsiung City, Taiwan. We conducted this study using a case–control study design by matching age and class exposure. Concentrations of airborne bacteria and fungi in homes were also evaluated, as well as temperature and relative humidity; moreover, the relationship between household characteristics and these biological contaminants was also explored.

## Materials and methods

### Study design and population

In January 2010, cases and controls were recruited by distributing questionnaires to all school-age children attending grades 1–6 in three elementary schools located in northern, central, and southern parts of Kaohsiung City, respectively. Kaohsiung City (22 N 38′, 120 E^.^17′), located in southern Taiwan, is the largest industrialized harbor city in Taiwan. Dense industrial activities and traffic have made Kaohsiung City, the surrounding Kaohsiung County, and Pingtung County have the poorest air quality in Taiwan. The total population of Kaohsiung City was approximately 1.52 million people in 2010.

Case and control status was defined by a health assessment at schools including an International Study of Asthma and Allergies in Childhood (ISAAC) questionnaire-standardized interview (Asher et al. 1995). Cases comprised subjects positively responding to the question “Has a doctor ever diagnosed that your son or daughter had asthma and the allergy.” Controls for each case were selected from each case’s classmates who were not considered cases. Cases and controls were exposed to the same air conditions in school during the school day. All cases and controls were eligible for the study with some exceptions. If a family had a school-age participant selected to be a case, other children in that family were not considered for selection as cases or controls. If more than one child in the family was a potential case, one case was randomly selected for participation. Moreover, case and control status was also confirmed by allergen testing and a clinical examination by a panel of respiratory pediatricians through the International European Respiratory Society/American Thoracic Society guidelines. Finally, 60 children with asthma, 30 children with allergy, and 60 healthy children voluntarily participated.

### Home visit

Data collection of home visits for this study included an interviewer-administered questionnaire (see online supplementary Table S1), air sampling of participants’ homes for endotoxin, bacteria and fungi, as well as temperature and relative humidity measurements. Environmental sampling consisted of two visits per home, 24 h apart. These visits were conducted by the sampling team blind to the case–control status of the household. On the first visit, a survey questionnaire about house characteristics was administered, and bacterial and fungal bioaerosols were evaluated. Then, airborne endotoxin sampling cassettes were left and sampled for 24 h. On the second visit, the endotoxin cassettes were collected, and bacterial and fungal bioaerosols were evaluated again.

### Sampling and analysis of airborne endotoxin

The sampling, extraction method and standard curves of indoor airborne endotoxin were derived as described in detail by Yen et al. (2019). In brief, before sampling, filters and support pads were autoclaved, and the standard-style 37- mm three-piece cassettes (Cat. No. 225-3050 LF, SKC Inc., Texas, USA) were sterilized with ethylene oxide. Airborne endotoxin was collected on 1-μm pore size of polytetrafluoroethylene (PTFE; Teflon) membrane filters in the disposable plastic cassettes by using a sampling pump operating at 20 L m^−1^ with a sampling time of 24 h. Once collected, the samples were sealed in new plastic bags and then transported at 4 °C to our laboratory (Kaohsiung City, Taiwan, Republic of China) within 1 h. The sampling height was 0.55 m above the floor near children’s beds in the bedroom to stimulate the children’s breath zone when they were sleeping. For quality control, trip blank and field blank were also evaluated. Results confirmed no detectable endotoxin in either trip blanks or field blank (data not shown). In addition, side-by-side duplicate field samples yielded comparable results (with relative difference of 8%). The extraction and analysis of airborne endotoxin were described in supplementary manuscript. We accepted the data only when *R*^2^ was greater than 0.9, and all of the negative controls were negative.

### Bacterial and fungal bioaerosols

Triplicate total cultivable airborne bacteria and fungi samples were collected using a portable microbiological air sampler (MAS-100; MERCK, USA). The tryptic soy agar (TSA, Difco Laboratories, Michigan, USA) and malt extract agar (MEA, Difco Laboratories, Michigan, USA) were used for bacteria and fungi, respectively. The bacteria were incubated at 37 °C for 24 to 48 h, and fungi were incubated at 25 °C for 48 to 72 h (Chi and Li 2007). The concentrations were expressed as colony-forming units per cubic meter of air (CFU m^−3^). For each sampling, the air sampler was always wiped with alcohol between sample collections to avoid cross-contamination. Two field blanks were assessed for every 10 households and no field blank samples was positive.

### Statistical analysis

SAS statistical package version 9.3 (SAS Institute Taiwan Ltd) was used for analysis. Because the concentrations of airborne bacteria, fungi, and endotoxin were not normally distributed (data not shown), we analyzed our data by nonparametric statistics, also known as distribution-free statistics. The *P* value for difference in proportions and median by case–control status was calculated using the Fisher exact test and the Wilcoxon rank sum test, respectively. In addition, these pollutants were also analyzed using quartile-defined categories. The logistic regression was used to assess the associations of airborne endotoxin, bacteria, and fungi with case–control status, sinusitis, wheeze, and allergic rhinitis, etc. Odds ratios (ORs) of airborne endotoxin, bacteria, and fungi and the corresponding 95% confidence intervals (CIs) for the associations were reported for Q2 versus the lowest endotoxin quartile(Q1), for Q3 versus Q1, and for Q4 (the highest quartile) versus Q1. For evaluating the correlation between airborne endotoxin, bacteria, and fungi and case–control status, gender, father’s education, mother’s education, keeping pets, and smokers at home were adjusted. For evaluating the correlation between airborne endotoxin, bacteria, and fungi and respiratory symptoms, gender, father’s education, mother’s education, keeping pets, smokers at home, and case–control status were adjusted. Due to the extreme values of airborne endotoxin, bacteria, and fungi concentrations in our study, the robust regression analysis was utilized to assess the associations between airborne endotoxin, bacteria, and fungi concentrations and the temperature, relative humidity, and parameters of household characteristics. A *P* value of less than 0.05 was considered significant.

## Results

Nearly 2000 (1934) children were eligible for the study after they responded to a validated screening questionnaire. Only 449 of households agreed to take part in the study. Because the children had to be matched based on classroom and availability for a visit within the same week, only 120 children were finally recruited into the study and completed the whole sampling process. From April 2010 to February 2012, 60 asthmatic and allergic children and 60 matched healthy classmates were recruited and completed the process. The demographic characteristics, health conditions, and parental disease of the study participants were described in Table s1. There was no significant difference between case and control group in the percentage of gender, parental education, smoker, and furry pet at home, health condition (such as sinusitis, wheeze, etc.) of children and parents. Table 1 shows the descriptive statistics of concentrations of airborne endotoxin, bacteria, and fungi, as well as temperature and relative humidity. For airborne endotoxin, bacteria, and fungi concentrations, there was no statistically significant difference of between case and control groups (see online supplementary Table s2).

**Table 1:** Descriptive statistics of airborne endotoxin, bacteria, and fungi concentration, temperature, and relative humidity

	Mean	Standard deviation	25th percentile	Median (50th percentile)	75th percentile	Minimum	Maximum
Airborne endotoxin (EU m^−3^)	1.40	0.16	0.31	0.67	1.97	0.02	8.13
Airborne bacteria (CFU m^−3^)	1771	156	557	1411	2468	129	7008
Airborne fungi (CFU m^−3^)	548	61	159	314	699	44	4014
Temperature (°C)	30.63	0.20	29.51	31.13	31.89	23.30	34.33
Relative humidity (%)	72.26	0.47	69.22	72.09	75.84	60.48	83.67

Tables 2, 3, and 4 demonstrated the associations between children’s health effects and airborne endotoxin, bacteria, and fungi, respectively. In the adjusted logistic regression model, household airborne endotoxin was associated with higher prevalence of asthma and allergy; OR = 4.88 (95% CI 1.16–20.55) for Q3 (between 0.67 and 1.97 EU m^−3^) vs. Q1 (< 0.31 EU m^−3^) (Table 2). In both unadjusted and adjusted logistic regression models, airborne bacteria was associated with lower prevalence of allergic eczema for Q3 (between 1411 and 2468 CFU m^−3^) vs. Q1 (< 557 CFU m^−3^) (OR = 0.16 (95% CI 0.03–0.96) and 0.10 (95% CI 0.01–1), respectively) (Table 3). In both unadjusted and adjusted logistic regression models, airborne fungi was associated with higher prevalence of asthma and allergy for Q3 (between 314 and 699 EU m^−3^) vs. Q1 (< 159 CFU m^−3^) (OR = 3.53 (95% CI 1.27–9.80) and OR = 4.47 (95% CI 1.13–17.69), respectively) (Table 4).

**Table 2:** Odds ratios (ORs) for association of airborne endotoxin with respiratory disease and symptoms

Airborne endotoxin exposure^a^		Crude OR (95% CI^b^)	*P* value	Adjusted OR(95% CI^b^)	*P* value
Have been diagnosed					
Asthma and allergy	Q2 vs. Q1	0.49 (0.18–1.35)	0.17	0.73 (0.15–3.49)	0.70
	Q3 vs. Q1	2.05 (0.73–5.74)	0.17	4.88 (1.16–20.55)*	0.03
	Q4 vs. Q1	0.51 (0.19–1.37)	0.18	0.70 (0.16–3.06)	0.64
Sinusitis	Q2 vs. Q1	0.71 (0.16–3.11)	0.65	0.99 (0.19–5.17)	0.99
	Q3 vs. Q1	0.46 (0.13–1.64)	0.23	0.57 (0.14–2.37)	0.44
	Q4 vs. Q1	1.14 (0.24–5.42)	0.87	1.30 (0.25–6.86)	0.76
Wheeze	Q2 vs. Q1	1.09 (0.23–5.16)	0.91	1.14 (0.20–6.36)	0.88
	Q3 vs. Q1	0.76 (0.20–2.98)	0.70	0.57 (0.12–2.58)	0.46
	Q4 vs. Q1	0.82 (0.19–3.48)	0.79	0.66 (0.14–3.07)	0.59
Allergic rhinitis	Q2 vs. Q1	0.37 (0.09–1.53)	0.17	0.25 (0.05–1.26)	0.09
	Q3 vs. Q1	0.37 (0.11–1.26)	0.11	0.30 (0.07–1.25)	0.10
	Q4 vs. Q1	0.92 (0.22–3.92)	0.91	0.81 (0.17–3.82)	0.79
Allergic eczema	Q2 vs. Q1	0.47 (0.08–2.83)	0.41	0.44 (0.06–3.33)	0.42
	Q3 vs. Q1	3.00 (0.29–31.48)	0.36	2.21 (0.17–29.64)	0.55
	Q4 vs. Q1	0.58 (0.10–3.38)	0.54	0.55 (0.06–5.36)	0.61
Bronchitis	Q2 vs. Q1	1.23 (0.35–4.34)	0.75	1.54 (0.38–6.22)	0.54
	Q3 vs. Q1	1.26 (0.42–3.77)	0.68	1.34 (0.39–4.57)	0.64
	Q4 vs. Q1	1.48 (0.45–4.90)	0.52	1.92 (0.52–7.16)	0.33
Pneumonia	Q2 vs. Q1	1.31 (0.22–7.88)	0.77	5.55 (0.44–69.89)	0.19
	Q3 vs. Q1	1.43 (0.30–6.82)	0.65	0.90 (0.14–5.92)	0.91
	Q4 vs. Q1	1.03 (0.21–5.06)	0.97	1.29 (0.20–8.38)	0.79
In the past 12 months					
Sinusitis	Q2 vs. Q1	0.28 (0.05–1.53)	0.14	0.34 (0.03–3.59)	0.37
	Q3 vs. Q1	0.36 (0.08–1.69)	0.19	0.27 (0.04–1.79)	0.18
	Q4 vs. Q1	2.22 (0.22–22.70)	0.50	5.77 (0.23–143.42)	0.28
Wheeze	Q2 vs. Q1	0.16 (0.02–1.28)	0.08	2.86 (0.03–311.76)	0.66
	Q3 vs. Q1	0.30 (0.04–2.27)	0.24	0.21 (0.02–2.51)	0.22
	Q4 vs. Q1	0.51 (0.068–4.43)	0.54	1.18 (0.08–17.99)	0.90

^a^*Q1*, 1st quartile < 0.31 EU m^−3^; *Q2*, 2nd quartile 0.31–0.67 EU m^−3^; *Q3*, 3rd quartile 0.67–1.97 EU m^−3^; *Q4*, 4th quartile > 1.97 EU m^−3^

^b^95% confidence intervals

**Table 3:** Odds ratios (ORs) for association of airborne bacteria with respiratory disease and symptoms

Airborne bacteria exposure^a^		Crude OR (95% CI^b^)	*P* value	Adjusted OR (95% CI^b^)	*P* value
Have been diagnosed					
Asthma and allergy	Q2 vs. Q1	0.99 (0.37–2.62)	0.98	1.58 (0.44–5.70)	0.49
	Q3 vs. Q1	1.46 (0.54–3.94)	0.46	0.72 (0.18–2.81)	0.63
	Q4 vs. Q1	1.34 (0.51–3.56)	0.56	1.15 (0.28–4.65)	0.85
Sinusitis	Q2 vs. Q1	1.58 (0.40–6.15)	0.51	1.78 (0.36–8.69)	0.48
	Q3 vs. Q1	0.99 (0.27–3.70)	0.99	1.89 (0.38–9.36)	0.43
	Q4 vs. Q1	1.65 (0.37–7.39)	0.51	2.07 (0.38–11.25)	0.40
Wheeze	Q2 vs. Q1	0.67 (0.16–2.92)	0.60	0.59 (0.12–3.02)	0.52
	Q3 vs. Q1	0.26 (0.06–1.07)	0.06	0.25 (0.05–1.18)	0.08
	Q4 vs. Q1	0.77 (0.16–3.76)	0.75	0.59 (0.11–3.19)	0.54
Allergic rhinitis	Q2 vs. Q1	1.77 (0.45–6.98)	0.42	1.76 (0.39–7.89)	0.46
	Q3 vs. Q1	0.52 (0.14–1.85)	0.31	0.65 (0.17–2.52)	0.53
	Q4 vs. Q1	1.06 (0.28–4.06)	0.93	0.98 (0.23–4.18)	0.98
Allergic eczema	Q2 vs. Q1	0.38 (0.06–2.56)	0.32	0.57 (0.05–6.63)	0.65
	Q3 vs. Q1	0.16 (0.03–0.96)*	0.04	0.10 (001–1.0)*	0.05
	Q4 vs. Q1	–	0.95	–	0.97
Bronchitis	Q2 vs. Q1	1.49 (0.45–4.89)	0.52	1.48 (0.39–5.57)	0.57
	Q3 vs. Q1	0.87 (0.25–2.96)	0.82	0.76 (0.20–2.89)	0.69
	Q4 vs. Q1	1.44 (0.41–5.07)	0.57	1.33 (0.33–5.37)	0.69
Pneumonia	Q2 vs. Q1	1.14 (0.24–5.49)	0.87	1.77 (0.30–10.39)	0.53
	Q3 vs. Q1	0.99 (0.20–4.82)	0.99	2.35 (0.30–18.34)	0.42
	Q4 vs. Q1	1.48 (0.25–8.73)	0.67	1.13 (0.16–7.86)	0.90
In the past 12 months					
Sinusitis	Q2 vs. Q1	–	0.96	–	0.96
	Q3 vs. Q1	1.40 (0.29–6.83)	0.68	0.51 (0.05–5.91)	0.59
	Q4 vs. Q1	0.47 (0.09–2.43)	0.37	0.13 (0.01–1.55)	0.11
Wheeze	Q2 vs. Q1	3.75 (0.33–43.28)	0.29	3.09 (0.15–63.44)	0.46
	Q3 vs. Q1	0.56 (0.09–3.52)	0.54	0.81 (0.09–6.99)	0.85
	Q4 vs. Q1	2.63 (0.22–31.35)	0.45	2.46 (0.17–35.21)	0.51

^a^*Q1*, 1st quartile < 577 CFU m^−3^; *Q2*, 2nd quartile 557–1411 CFU m^−3^; *Q3*, 3rd quartile 1411–2468 CFU m^−3^; *Q4*, 4th quartile > 24,687 CFU m^−3^

^b^95% confidence interval

**Table 4:** Odds ratios (ORs) for association of airborne fungi with respiratory disease and symptoms

Airborne fungi exposure^a^		Crude OR (95% CI^b^)	*P* value	Adjusted OR (95% CI^b^)	*P* value
Have been diagnosed					
Asthma and allergy	Q2 vs. Q1	1.87 (0.69–5.03)	0.22	1.86 (0.43–7.93)	0.40
	Q3 vs. Q1	3.53 (1.27–9.80)*	0.02	4.47 (1.13–17.69)*	0.03
	Q4 vs. Q1	2.80 (1.01–7.74)*	0.05	2.91 (0.75–11.27)	0.12
Sinusitis	Q2 vs. Q1	1.54 (0.39–6.08)	0.54	1.53 (0.27–8.57)	0.63
	Q3 vs. Q1	1.04 (0.28–3.91)	0.95	1.47 (0.31–6.87)	0.63
	Q4 vs. Q1	2.05 (0.47–9.06)	0.34	2.08 (0.41–10.69)	0.38
Wheeze	Q2 vs. Q1	0.48 (0.13–1.70)	0.26	0.44 (0.10–2.02)	0.29
	Q3 vs. Q1	3.96 (0.44–35.81)	0.22	4.84 (0.51–45.64)	0.17
	Q4 vs. Q1	1.32 (0.29–5.99)	0.72	1.54 (0.30–7.85)	0.60
Allergic rhinitis	Q2 vs. Q1	1.10 (0.28–4.27)	0.89	0.89 (0.18–4.45)	0.89
	Q3 vs. Q1	1.15(0.32–4.08)	0.83	1.19 (0.30–4.71)	0.81
	Q4 vs. Q1	1.51 (0.41–5.59)	0.53	1.96 (0.47–8.29)	0.36
Allergic eczema	Q2 vs. Q1	0.43 (0.09–2.10)	0.30	0.77 (0.07–7.92)	0.82
	Q3 vs. Q1	2.86 (0.29–28.17)	0.37	3.19 (0.27–37.40)	0.36
	Q4 vs. Q1	2.48 (0.25–24.64)	0.44	4.08 (0.28–60.54)	0.31
Bronchitis	Q2 vs. Q1	1.83 (0.53–6.34)	0.34	1.56 (0.37–6.57)	0.54
	Q3 vs. Q1	1.00 (0.31–3.27)	1.00	0.90 (0.24–3.39)	0.88
	Q4 vs. Q1	2.20 (0.61–8.00)	0.23	2.07 (0.53–8.15)	0.30
Pneumonia	Q2 vs. Q1	0.99 (0.20–4.82)	0.99	1.03 (0.15–7.26)	0.98
	Q3 vs. Q1	0.74 (0.17–3.25)	0.69	0.87 (0.15–4.96)	0.88
	Q4 vs. Q1	3.41 (0.36–32.19)	0.28	3.34 (0.30–36.86)	0.32
In the past 12 months					
Sinusitis	Q2 vs. Q1	1.46 (0.31–6.98)	0.64	3.18 (0.16–63.94)	0.45
	Q3 vs. Q1	1.02 (0.20–5.15)	0.98	0.10 (0.004–2.96)	0.18
	Q4 vs. Q1	3.50 (0.37–33.55)	0.28	–	0.05
Wheeze	Q2 vs. Q1	1.09 (0.15–7.80)	0.93	1.73 (0.15–19.93)	0.66
	Q3 vs. Q1	1.27 (0.18–8.89)	0.81	4.60 (0.23–93.01)	0.32
	Q4 vs. Q1	2.55 (0.23–27.71)	0.44	11.58 (0.44–305.03)	0.14

^a^*Q1*, 1st quartile < 159 CFU m^−3^; *Q2*, 2nd quartile 159–314 CFU m^−3^; *Q3*, 3rd quartile 314–699 CFU m^−3^; *Q4*, 4th quartile > 699 CFU m^−3^

^b^95% confidence interval

With regression analysis of the associations between airborne endotoxin concentrations and characteristics of the indoor environment (see online supplementary Table s3), higher endotoxin concentrations in the bedroom air were significantly associated with fragrance using. In addition, compared with the home without carpets, there were higher airborne bacteria concentrations in the home with carpets.

## Discussion

To our knowledge, this is the first study revealing the association between airborne endotoxin concentration and asthma and allergy status of children. So far, there were only eight studies exploring the association between airborne endotoxin concentrations (home/school/personal exposure) and respiratory health effects of children (Dales et al. 2006; Delfino et al. 2015; Hoopmann et al. 2006; Horick et al. 2006; Lai et al. 2015; Matsui et al. 2013; Rabinovitch et al. 2005; Ramagopal et al. 2014). The subjects in five of them were asthmatic schoolchildren (Delfino et al. 2015; Hoopmann et al. 2006; Lai et al. 2015; Matsui et al. 2013; Rabinovitch et al. 2005), and three of them were infant (Dales et al. 2006; Horick et al. 2006; Ramagopal et al. 2014). This is the first study to investigate the household airborne endotoxin concentration in asthmatic/allergic children (case group) and non-asthmatic and allergic children (control group).

In our study, the percentage of wheeze and eczema in the case (30.23% and 19.44%, respectively) were higher than in the control (14.29% and 9.38%, respectively), although there were no statistically significant differences. A small sample size (*N* = 120) might be the reason for the results.

In regard to the risk of airborne endotoxin on asthma and allergy status, we found that the highest prevalence of asthma/allergy was observed in the third quartile of household airborne endotoxin (between 0.67 and 1.97 EU m^−3^) with OR 4.88 when compared with the first quartile, and this result was consistent with previous studies. There was one study that revealed the highest risk of asthma and allergy was observed in Q3 dust endotoxin exposure concentrations (OR = 1.39 (95% CI 1.09–1.77) for Q2 vs. Q1; OR = 1.58 (95% CI 1.24–2.01) for Q3 vs. Q1; OR = 1.44 (95% CI 1.13–1.84) for Q4 vs. Q1) (Baker 2006). In addition, another study also found that the highest risk of children’s asthma was found in the 2nd tertiles airborne endotoxin exposure concentrations (OR = 2.6 (95% CI 0.6–11.3) for 2nd tertiles vs. 1st tertiles; OR = 1.7 (95% CI 0.3–10.0) for 3rd tertiles vs. 1st tertiles) (Ramagopal et al. 2014). However, the median airborne endotoxin concentration in control group (0.87 EU m^−3^) was higher than that of the case group (0.64 EU m^−3^) and the maximum concentration (8.13 EU m^−3^) was found in the control group. Low cleaning frequency in the control group might be the reason. In addition, it was found that that airborne endotoxin increases the risk of respiratory health in asthmatic schoolchildren in all previous studies (Delfino et al. 2015; Hoopmann et al. 2006; Lai et al. 2015; Matsui et al. 2013; Rabinovitch et al. 2005). Different study populations and different health outcomes might be the reasons for the differences between our study and previous studies.

In terms of airborne bacteria, our study displayed that there was an inverse association between airborne bacteria and allergic eczema in both univariate and multivariate analyses. According to a review study (Baker 2006), decreased exposure to microorganisms during early life may increase the prevalence and severity of atopic dermatitis and discussed the mechanisms. So far, only one study explored the relationship between airborne bacteria concentrations and allergic dermatitis (Kallawicha et al. 2016). They demonstrated that total bacteria were negatively associated with atopic dermatitis, contact dermatitis, and other eczema in univariate analysis, which was consistent with our results.

Regarding the airborne fungi, we found that airborne fungi were significantly associated with the higher prevalence of asthma or allergy, and this result was consistent with the observation in previous studies. It was found that the airborne fungi in allergic/asthmatic children’s house (the average annual concentration of total fungi was 110,091 CFU m^−3^) was significantly higher than that in healthy children’s house (the average annual concentration of total fungi was 107,850 CFU m^−3^) (Sharma et al. 2011). In addition, previous studies also demonstrated that indoor dampness/visible mold/mold odor heightened the incidence of asthma in children (Fisk et al. 2007; Hwang et al. 2011).

For environmental factors, we found that the use of fragrances was positively associated with airborne endotoxin. So far, no study has been conducted on the association between fragrances and endotoxin. Further exploration of the relationship between the use of fragrances and airborne endotoxin concentration will be needed for full understanding of the story. We also found that there were higher airborne bacteria and fungi concentrations in the home with carpets compared with the home without carpets, while only the results of airborne bacteria with statistical significance. Previously, there were many studies that investigated bacteria and fungi in household carpet (Karvonen et al. 2015; Macher 2001; Pekkanen et al. 2007) and they found that culturable bacteria and fungi could be isolated from carpet dust. Bacteria and fungi in carpet dust might be the source of indoor bacteria and fungi.

Our study has some limitations. Firstly, the health outcome in our study only focused on case–control status and did not discuss the severity of the disease. Secondly, the present study was a case–control study design, not a longitudinal study, so it hardly confirms a causal relationship between airborne endotoxin and asthma and allergy status. Thirdly, small sample sizes limited the conclusion and increase potential biases related to these data.

## Conclusion

In conclusion, the highest prevalence of asthma/allergy was observed in the third quartile of household airborne endotoxin (between 0.67 and 1.97 EU m^−3^) and fungi (between 314 and 699 CFU m^−3^) with OR of 4.88 (95% CI 1.16–20.55) and 4.47 (95% CI 1.13–17.69), respectively.

## Electronic supplementary material

ESM 1 (DOCX 25 kb).

ESM 2 (DOCX 12 kb).
