# Supplementary material for: Household airborne endotoxin associated with asthma and allergy in elementary school-age children: a case–control study in Kaohsiung, Taiwan
Source: Environ Sci Pollut Res Int. 2020 Mar 25;27(16):19502–9. doi: 10.1007/s11356-020-07899-x (PMC7244453; doi:10.1007/s11356-020-07899-x)
Supplement: Supplementary file 1 — (DOCX 25 kb). [file 11356_2020_7899_MOESM1_ESM.docx]

Table s1. Characteristics of the school-children of case-control groups (N=120)

|  | **All** | **Case** | | **Control** | | ***P*- value** |
| --- | --- | --- | --- | --- | --- | --- |
| **Age (year)** | 10.73 | | 10.66 | | 10.79 | 0.58 |
| **Gender (%)** |  | | | | | 0.39 |
| **Males** | 57.30 | 52.17 | | 62.79 | |  |
| **Females** | 42.70 | 47.83 | | 37.21 | |  |
| **Father's education (%)** | | | | | | 0.18 |
| **Junior High School** | 3.53 | 6.98 | | 0.00 | |  |
| **Senior High School** | 22.35 | 20.93 | | 23.81 | |  |
| **University** | 54.12 | 58.14 | | 50.00 | |  |
| **Master** | 20.00 | 13.95 | | 26.19 | |  |
| **Mother's education (%)** | | | | | | 0.45 |
| **Junior High School** | 3.66 | 4.76 | | 2.50 | |  |
| **Senior High School** | 39.02 | 38.10 | | 40.00 | |  |
| **University** | 50.00 | 54.76 | | 45.00 | |  |
| **Master** | 6.10 | 2.38 | | 10.00 | |  |
| **Two-year Junior College** | 1.22 | 0.00 | | 2.50 | |  |
| **Smokers (%)** | | | | | | 0.82 |
| **Yes** | 36.78 | 34.78 | | 39.02 | |  |
| **No** | 63.22 | 65.22 | | 60.98 | |  |
| **Furry pet (%)** | | | | | | 0.27 |
| **Yes** | 20.48 | 15.22 | | 27.03 | |  |
| **No** | 79.52 | 84.78 | | 72.97 | |  |
| **The number of colds in a year (%)** | | | | | | 0.77 |
| **0** | 6.10 | 4.76 | | 7.50 | |  |
| **1-3** | 58.54 | 54.76 | | 62.50 | |  |
| **4-5** | 24.39 | 28.57 | | 20.00 | |  |
| **6** | 10.98 | 11.90 | | 10.00 | |  |
| **Have been diagnosed by a physician** | | | | | | |
| **Sinusitis (%)** | | | | | | 0.80 |
| **Yes** | 27.27 | 25.00 | | 29.73 | |  |
| **No** | 72.73 | 75.00 | | 70.27 | |  |
| **Wheeze (%)** | | | | | | 0.11 |
| **Yes** | 23.08 | 30.23 | | 14.29 | |  |
| **No** | 76.92 | 69.77 | | 85.71 | |  |
| **Allergic rhinitis (%)** | | | | | | 0.47 |
| **Yes** | 37.50 | 42.11 | | 32.35 | |  |
| **No** | 62.50 | 57.89 | | 67.65 | |  |
| **Allergic eczema (%)** | | | | | | 0.31 |

| **Yes** | 14.71 | 19.44 | 9.38 |  |
| --- | --- | --- | --- | --- |
| **No** | 85.29 | 80.56 | 90.63 |  |
| **Bronchitis (%)** | | | | 0.50 |
| **Yes** | 43.04 | 47.50 | 38.46 |  |
| **No** | 56.96 | 52.50 | 61.54 |  |
| **Pneumonia (%)** | | | | 0.76 |
| **Yes** | 17.11 | 19.51 | 14.29 |  |
| **No** | 82.89 | 80.49 | 85.71 |  |
| **In the past 12 months** | | | | |
| **Sinusitis (%)** | | | | 0.76 |
| **Yes** | 25.45 | 23.33 | 28.00 |  |
| **No** | 74.55 | 76.67 | 72.00 |  |
| **Wheeze (%)** | | | | 0.12 |
| **Yes** | 22.50 | 14.81 | 38.46 |  |
| **No** | 77.50 | 85.19 | 61.54 |  |
| **Father’s disease** | | | | |
| **Asthma (%)** | | | | - |
| **Yes** | 0 | 0 | 0 |  |
| **No** | 100 | 100 | 100 |  |
| **Allergic rhinitis (%)** | | | | 0.65 |
| **Yes** | 34.12 | 37.21 | 30.95 |  |
| **No** | 65.88 | 62.79 | 69.05 |  |
| **Allergic eczema (%)** | | | | 0.20 |
| **Yes** | 7.32 | 2.44 | 12.20 |  |
| **No** | 92.68 | 97.56 | 87.80 |  |
| **Mother’s disease** | | | | |
| **Asthma (%)** | | | | 0.12 |
| **Yes** | 4.88 | 9.30 | 0.00 |  |
| **No** | 95.12 | 90.70 | 100.00 |  |
| **Allergic rhinitis (%)** | | | | 0.57 |
| **Yes** | 17.86 | 20.93 | 14.63 |  |
| **No** | 82.14 | 79.07 | 85.37 |  |
| **Allergic eczema (%)** | | | | 0.22 |

Table s2. Airborne endotoxin, bacteria, and fungi concentration, temperature, and relative humidity of case and control group

|  | **Case** | | **Control** | | ***P*- value** |
| --- | --- | --- | --- | --- | --- |
|  | Median± SE. | Range | Median±SE. | Range |  |
| **Airborne endotoxin ( EUm-3)** | 0.64±0.23 | 0.04-8.01 | 0.87±0.23 | 0.02- 8.13 | 0.20 |
| **Airborne bacteria****( CFUm-3)** | 1411±246 | 219- 7008 | 1400±200 | 129- 6669 | 0.59 |
| **Airborne fungi ( CFUm-3)** | 227±106 | 44- 4014 | 369±70 | 53- 2337 | 0.14 |
| **Temperature (℃)** | 31.10±0.24 | 25.12-34.33 | 31.31±0.31 | 23.30-33.69 | 0.77 |
| **Relative humidity (%)** | 71.76±0.65 | 60.48- 83.67 | 73.16±0.66 | 62.13- 82.46 | 0.11 |

Table s3. Association between bioaerosols and the environmental parameters: the robust regression analysis.

| **Environmental parameter** | **Airborne endotoxin** | | **Airborne bacteria** | | **Airborne fungi** | |
| --- | --- | --- | --- | --- | --- | --- |
|  | β±SE. | *P*-value | β±SE. | *P*-value | β±SE. | *P*-value |
| **Temperature** | -0.09±0.06 | 0.12 | 57±67 | 0.39 | -29±16 | 0.07 |
| **Relative Humidity** | -0.02±0.02 | 0.37 | -39±27 | 0.15 | 7±7 | 0.28 |
| **Airborne endotoxin** | - | - | -30±79 | 0.71 | -5±21 | 0.80 |
| **Airborne bacteria** | -0.0001±0.0001 | 0.08 | - | - | -0.0127±0.0212 | 0.55 |
| **Airborne fungi** | 0.0002±0.0002 | 0.20 | -0.0432±0.2123 | 0.84 | - | - |
| **Housing type**  **(Apartment vs. Townhouse)** | 0.05±0.43 | 0.91 | -342±379 | 0.37 | 72±112 | 0.52 |
| **House of age**  **(≥10 years vs. <10years)** | -0.10±0.41 | 0.80 | -6±367 | 0.99 | -98±109 | 0.37 |
| **Number of occupants**  **(> 4people vs. ≤ 4 people)** | 0.20±0.40 | 0.62 | 99±365 | 0.79 | 53±108 | 0.63 |
| **Type of adjacent road**  **(Multiple-ways vs. One-way)** | 0.55±0.41 | 0.18 | -324±403 | 0.42 | -184±119 | 0.13 |
| **Carpet (Yes vs. No)** | 0.07±1.17 | 0.95 | **2110±1065*** | **0.05** | 198±334 | 0.55 |
| **Furry pet (Yes vs. No)** | 0.82±0.44 | 0.07 | -561±415 | 0.18 | 151±129 | 0.24 |
| **Smoker (Yes vs. No)** | 0.36±0.42 | 0.39 | 56±374 | 0.88 | 7±113 | 0.95 |
| **Burning incense (Yes vs. No)** | 0.18±0.23 | 0.43 | 22.99±290.53 | 0.94 | -91.92±96.54 | 0.34 |
| **Visible mold (Yes vs. No)** | -0.26±0.40 | 0.52 | -420±377 | 0.27 | 119±114 | 0.30 |
| **Air Purifier (Yes vs. No)** | 0.50±0.42 | 0.23 | -86.±387 | 0.82 | 44±115 | 0.70 |
| **Dehumidifier (Yes vs. No)** | 0.58±0.43 | 0.19 | 141±396 | 0.72 | 25±118 | 0.83 |
| **Vacuum cleaner (Yes vs. No)** | 0.61±0.39 | 0.13 | -303±365 | 0.41 | 79±108 | 0.47 |
| **Fragrance (Yes vs. No)** | **1.89±0.44*** | **<.0001** | -131±442 | 0.77 | 66±131 | 0.62 |
| **Essential oils (Yes vs. No)** | -0.18±0.57 | 0.75 | -80±511 | 0.88 | -99±147 | 0.50 |
| **Fluffy toys(Yes vs. No)** | 0.06±0.45 | 0.89 | -52±403 | 0.90 | -188±118 | 0.11 |
