# Supplementary material for: Household airborne endotoxin associated with asthma and allergy in elementary school-age children: a case–control study in Kaohsiung, Taiwan
Source: Environ Sci Pollut Res Int. 2020 Mar 25;27(16):19502–9. doi: 10.1007/s11356-020-07899-x (PMC7244453; doi:10.1007/s11356-020-07899-x)
Supplement: Supplementary file 2 — (DOCX 12 kb). [file 11356_2020_7899_MOESM2_ESM.docx]

**Extraction and analysis of airborne endotoxin**

The aerosol samples were extracted with 1000 µl of non-pyrogenic water which contained 0.005% tween 20, and centrifuged at 1000 rpm for five minutes. The supernatants of samples were analysed for endotoxin. The elute was then assayed with the Limulus Amebocyte Lysate kit, a US Food and Drug Administration–standardized bioassay for endotoxin (QCL-1000; Bio-Whittaker, Walkersville, Md, USA). The standard curves were made by reconstituting the endotoxin standard Escherichia coli O55: B5 with non-pyrogenic water, with the concentration range of 0.005 EU ml -1 to 50 EU ml -1. Finally, the samples were analysed by Enzyme-linked immunosorbent assay (ELISA, PowerWave XS2, BioTek Instruments, Inc., USA) and the fluorescent wavelength was 405 nm. We accepted the data only when R^2^ was greater than 0.9, and all of the negative controls were negative.
